# Aberrant ICA in the middle ear

**DOI:** 10.1007/s00405-026-10500-9

**Published:** 2026-08-03

**Authors:** Mugurel Constantin Rusu

**Affiliations:** Division of Anatomy, Faculty of Dentistry, “Carol Davila” University of Medicine and Pharmacy, Bucharest, 020021 Romania

**Keywords:** Aberrant internal carotid artery, Middle ear, Pulsatile tinnitus, Carotid canal dehiscence, Myringotomy, Iatrogenic vascular injury, High-resolution computed tomography, Lateralised petrous internal carotid artery

## Abstract

An aberrant internal carotid artery (a-ICA) in the middle ear is rare, but it may be injured during routine otological procedures. This review examines 48 publications on its embryological basis, clinical presentation, imaging diagnosis, and management. The a-ICA results from agenesis of the first embryonic segment of the internal carotid artery, with collateralisation through the inferior tympanic artery. Presentation is often non-specific; hearing loss (47.7%) and pulsatile tinnitus (30.0%) are the most frequent symptoms. Otoscopy may show a pulsatile retrotympanic mass in the anteroinferior quadrant. High-resolution computed tomography is central to diagnosis; relevant features include absence of the normal vertical carotid canal and its exocranial opening, and enlargement of the inferior tympanic canaliculus. The a-ICA must be distinguished from the more common lateralised petrous ICA variant, which retains a normal proximal calibre and enters the anterior mesotympanum without involving the inferior tympanic canaliculus. In iatrogenic injury, immediate packing is followed by definitive treatment in most reported cases; endovascular approaches have fewer complications than surgical ligation. Preoperative recognition remains one of the main safeguards against severe haemorrhage and neurological sequelae.

## Introduction

The aberrant ICA (a-ICA) in the middle ear is a rare vascular anomaly that can be hazardous during routine otological procedures, including myringotomy or tympanotomy. This review uses case reports and previous reviews [1–48] to summarise the aetiology, clinical presentation, imaging findings, and management of this condition, including the lateralised petrous ICA variant.

The risk arises from the vessel’s unusual, dehiscent, or displaced course into the tympanic cavity, where it is vulnerable to iatrogenic injury, with possible severe haemorrhage and neurological sequelae [4, 32, 41]. Recognition is important because it may mimic vascular tumours or other middle ear pathology, and intervention without appropriate imaging may be fatal [44]. When a pulsatile retrotympanic mass is investigated, high-resolution computed tomography (HRCT) should be assessed for features of an a-ICA [40].

## Aetiology, embryology, and classification

The a-ICA is generally regarded as a congenital collateral pathway that develops when the normal proximal ICA segment is absent.

A Embryological basis

Formation of an a-ICA has been attributed to segmental agenesis of the first embryonic segment (C1) of the internal carotid artery. During normal development, the ICA arises from the third aortic arch and dorsal aorta. If the cervical (C1) segment fails to develop, collateral channels maintain cerebral perfusion.

In this mechanism, the ascending pharyngeal artery, from the external carotid artery, enlarges and connects with the inferior tympanic artery. This vessel enters the middle ear through the inferior tympanic canaliculus, anastomoses with the caroticotympanic artery, a remnant of the hyoid artery, and joins the horizontal petrous ICA [44]. The resulting collateral is the clinically visible a-ICA. It enters the skull base posterior and lateral to the normal carotid canal, traverses the middle ear, and lacks the normal bony carotid plate. Rarely, two anomalous arteries may pass through the middle ear: an a-ICA and an inferior tympanic-to-petrous carotid anastomosis [40].

B Classification of middle ear vascular anomalies

Sullivan et al. (2019) classified middle ear vascular anomalies as arterial or venous. Among arterial anomalies, the a-ICA presents with a lateralised, dehiscent course through the middle ear and an enlarged inferior tympanic canaliculus. The persistent stapedial artery, another arterial anomaly, courses through the obturator foramen of the stapes and is often associated with a-ICA. Venous anomalies include a high jugular bulb, in which the jugular bulb extends superiorly into the middle ear and is often dehiscent. A dehiscent jugular bulb lacks the normal bony covering and is exposed to the middle ear [36].

C Prevalence and incidence

The exact incidence in the general population is unknown, and the available figures require caution: most series are small, heterogeneous in method, and likely affected by selection bias, so the true population prevalence is probably lower than the reported ranges. In these studies, available data suggest that the true a-ICA is extremely rare. At the same time, simple dehiscence of the carotid canal is more common. In the general population, based on imaging studies, Koesling (2005) found that a-ICA occurs in less than 1% of cases (specifically 0.2% in 446 temporal bones), while simple carotid canal dehiscence was identified in 2% to 7.7% of cases [19, 46]. In histological series, carotid canal dehiscence is far more frequent - 7.7% [46] - whereas computed tomography detects it in as few as 1.4% [48], reflecting modality rather than a true difference in incidence. In cohorts of patients presenting with pulsatile tinnitus, the incidence of a-ICA appears slightly higher, with Sonmez (2007) reporting 1.4% (1 of 74 patients) and Lv (2023) reporting 2.2% (1 of 45 patients with vascular aetiology) [24, 34]. These figures are approximate rather than definitive: the wide range likely reflects differences in case definition (true a-ICA versus simple carotid canal dehiscence), imaging modality (high-resolution CT versus older protocols), and study population (general radiological series versus selected pulsatile-tinnitus cohorts) rather than true differences in incidence.

## Clinical presentation

Symptoms of a-ICA and lateralised ICA often overlap clinically and may present with pulsatile tinnitus. Hearing loss is the most common symptom, reported in 47.7% of cases in Windfuhr’s review of 86 cases [41]. This hearing loss is most often conductive in nature, though mixed hearing loss and sensorineural hearing loss have also been reported [2, 6, 7, 9, 26, 33, 44]. The mechanism of sensorineural hearing loss may involve embryological entrapment leading to cochlear anomaly, since the medial wall of the cochlea is formed by the carotid canal, or the a-ICA may interfere with blood flow to the cochlea [44]. Additionally, bony dehiscence between the apex of the cochlea and the carotid artery may cause conductive hearing loss due to the “third window effect” [44]. Differential diagnoses for hearing loss include otosclerosis, serous otitis media, glomus tumour, and cholesteatoma.

Pulsatile tinnitus or audible bruit is another frequent symptom for both a-ICA and lateralised ICA, occurring in approximately 30% of a-ICA cases. Patients often describe an audible “bruit” or pulsations synchronous with heart rate [5, 8, 19, 20, 22, 24, 27, 34, 41, 44]. The differential diagnosis includes glomus tumours, high jugular bulb, and arteriovenous malformation.

Otoscopic examination may show a reddish, blue-reddish, pale red, or whitish pulsatile or nonpulsatile mass typically seen in the anteroinferior quadrant in cases of a-ICA, or in the anterior mesotympanum in cases of lateralised ICA [3, 7, 9, 19, 24, 26, 34, 41, 44]. Some parts of the tympanic membrane may appear attached to the mass without motion synchronous with pressure changes [44]. The differential diagnosis for such masses includes glomus tympanicum, dehiscent jugular bulb, and cholesterol granuloma.

Pain, including otalgia or postauricular pain, is less common but has been specifically reported as a rare symptom that may be treatable with medication [6, 41]. Vestibular symptoms, including unsteadiness, have also been reported [44]. Differential diagnoses include mastoiditis, temporomandibular joint disorders, and referred pain.

Clinically, a-ICA is often misdiagnosed as Ménière's disease, otitis media with effusion, or acute otitis media, potentially leading to inappropriate and dangerous surgical intervention [12, 15, 17, 19, 21, 32, 38, 41].

An a-ICA can coexist with other vascular anomalies, a point that matters when planning middle ear surgery. Associated anomalies include dehiscent high jugular bulb [12, 23], persistent stapedial artery [19, 22, 28], and carotid duplication [10, 25].

Rare complications include otic capsule erosion, in which an aberrant artery erodes the bony otic capsule into the cochlea, thereby allowing direct transmission of pulse vibrations [43]. This is confirmed by HRCT, which shows erosion into the inferior turn of the cochlea. Aneurysm or pseudoaneurysm formation can occur in either a-ICA or lateralised ICA, typically following trauma such as T-tube insertion or myringotomy [11, 13, 16, 17, 30, 38, 45]. These require surgical assessment due to the risk of rupture.

Kimura (2015) reported a fatal complication in which cerebral air embolism occurred after Eustachian tube air inflation due to a damaged, exposed arterial wall [18]. This case illustrates the vulnerability of a-ICA to pressure changes and non-surgical procedures.

## Diagnostic imaging features and distinctions

**Fig. 1:**
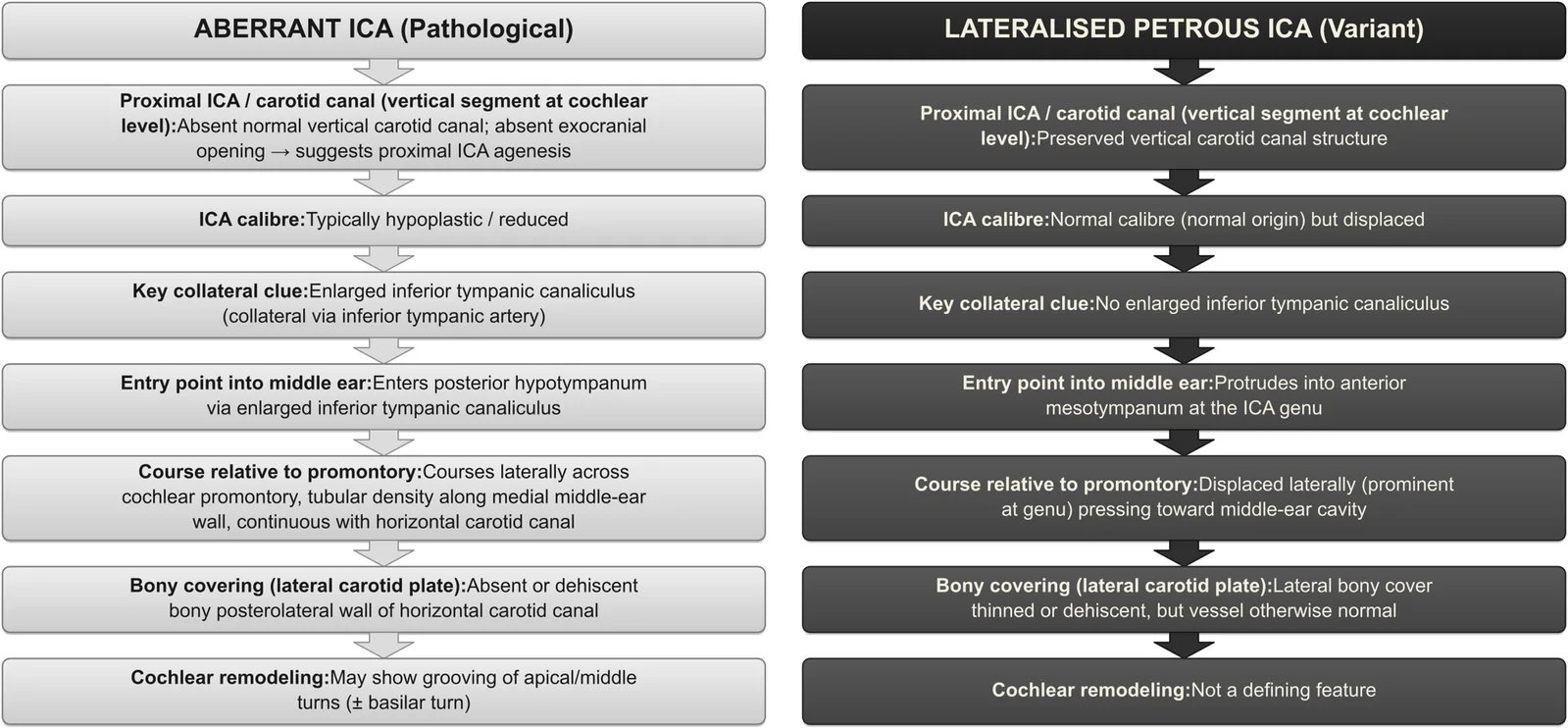
Distinguishing aberrant from lateralised internal carotid arteries (ICA)

Imaging is required to distinguish these entities (Fig. 1), because management and surgical risk depend on the exact course of the artery. Differential diagnosis before surgical manipulation is necessary for a pulsating mass behind the tympanic membrane [44]. HRCT defines the bony anatomy of the temporal bone and separates true anomalies from variants. Early work by Swartz et al. [37] and Willinsky et al. [40] established the CT criteria for a-ICA and demonstrated that CT could obviate the need for diagnostic angiography [37, 40]. Multislice CT (MSCT) with 3D reconstruction can depict the relationship between the aberrant vessel and the ossicular chain [21].

### Key HRCT features for aberrant ICA (pathological)

In the proximal segment, the vertical carotid canal at the level of the cochlea is usually absent, as is the exocranial opening of the carotid canal, indicating agenesis of the proximal ICA segment [11, 37, 40, 41, 44]. The calibre of the vessel itself, as visible on MRA, is usually hypoplastic or reduced [44]. Regarding the entry point and course, the artery enters the middle ear space in the posterior hypotympanum through a visibly enlarged inferior tympanic canaliculus (Jacobson’s canal), supporting a compensatory collateral pathway through the inferior tympanic artery [11, 26, 31, 33, 44]. The vessel then courses laterally across the cochlear promontory with a tubular density coursing along the medial wall of the middle ear in continuity with the horizontal carotid canal [40, 44]. Axial CT also shows the absence or dehiscence of the bony posterolateral wall (lateral carotid plate) of the horizontal portion of the carotid canal, with a hypotympanic soft tissue mass contiguous with this defect through a dehiscence of the lateral carotid plate [11, 37, 40, 41, 44]. There may be associated grooving of the apical turn, the middle turn, and to a lesser degree the basilar turn of the cochlea [37].

### Key HRCT features for lateralised petrous ICA (variant)

In the proximal segment, the vessel maintains a normal calibre, indicating a normal origin, but is displaced [11, 41]. Regarding the entry point and course, the ICA protrudes into the anterior mesotympanum (at the ICA genu) and does not involve an enlarged inferior tympanic canaliculus [11, 26, 31, 33]. The bony cover is either thinned or dehiscent at the lateral wall of the petrous ICA, allowing the displaced but otherwise normal vessel to press against the middle ear cavity. The vertical carotid canal structure is generally preserved [11, 26].

### MRI and MRA applications

Contrast-enhanced MRI helps differentiate vascular masses from neoplasms such as glomus tumour or cholesteatoma [26, 33, 41, 44]. MRA can confirm the vessel course, evaluate the calibre (hypoplastic in a-ICA, normal in lateralised ICA), detect pseudoaneurysm formation, and assess cerebral cross-circulation for surgical planning [11, 26, 33, 41, 44]. However, turbulent flow in aberrant courses can create a false appearance of stenosis due to a dephasing artefact [8]. Swartz et al. (1985) demonstrated that angiographic correlation with CT findings shows the ICA extending lateral to the vestibular line with focally reduced calibre at the site of bone deficiency [37].

## Case reports: spectrum of clinical presentations

Table 1 groups sixteen representative a-ICA cases reported over more than four decades. The cases include incidental findings, pulsatile tinnitus, hearing loss, postauricular pain and fatal complications; paediatric and adult patients; associated persistent stapedial artery, duplicated ICA and dehiscent jugular bulb; and management by observation, pharmacological symptom control, surgical separation, endovascular occlusion, hybrid bypass-and-trapping or ligation.

**Table 1:** Reports of cases of aberrant internal carotid arteries

Case/Year	Key Findings	Management	Citation
Goodman 1981	Iatrogenic pseudoaneurysm post-myringotomy; carotid canal posterior to normal location	Ligation (adequate cross-circulation)	[13]
Swartz 1985	Established CT diagnostic criteria; demonstrated absence of vertical carotid canal and cochlear grooving	Diagnostic imaging study	[37]
Jacobsson 1989	3-year-old with recurrent bleeding after myringotomy; missing bony wall	Prolonged packing (76 days)	[15]
Willinsky 1990	Unusual variation: TWO anomalous arteries (a-ICA + inferior tympanic to petrous carotid anastomosis)	Diagnostic imaging study	[40]
Yao 1998	Bilateral a-ICA with otic capsule erosion into the inferior cochlear turn	Conservative	[43]
Jain 2002	Spontaneous fistula rupture with 1 L haemorrhage; prior surgery	Emergency balloon occlusion	[16]
Roll 2003	5-year-old: bilateral a-ICAs, bilateral persistent stapedial arteries, bilateral duplicated ICAs	Conservative	[28]
Sauvaget 2006	Series of 16 cases; 4 with surgical injury; endovascular occlusion → hemiparesis in 1	Various (endovascular, conservative)	[31]
Glastonbury 2012	Distinction criteria: lateralised (genu, normal calibre) vs. aberrant (hypotympanum, hypoplastic)	Diagnostic classification	[11]
Yetiser 2015	23-year-old with sensorineural hearing loss, unsteadiness, tinnitus; misdiagnosed as Ménière's disease	Conservative with medication; symptom relief	[44]
Kimura 2015	Fatal cerebral air embolism after Eustachian tube insufflation; histology showed thinned tunica media	Fatal outcome	[18]
Takano 2016	Bilateral pseudoaneurysm post-myringotomy; high-flow bypass failed	Ligation → stroke with hemiparesis	[38]
Kawamura 2017	31-year-old with ruptured pseudoaneurysm post-myringotomy	Hybrid OR: bypass + endovascular trapping (no deficits)	[17]
Anagiotos 2019	Duplication variant: hypoplastic native ICA with wider aberrant collateral via ITA	Conservative	[3]
Chen 2022	39-year-old female with postauricular pain and mixed hearing loss	Oxcarbazepine for pain; conservative	[6]
Spörlein 2025	Multicenter series: 4 injury cases with varying outcomes and complications	Endovascular coiling, conservative	[35]

##  Management strategies and risks

Iatrogenic a-ICA injury requires immediate haemorrhage control, vascular imaging and, when needed, definitive repair. Spörlein [35] and Bathla [4] support haemorrhage control followed by definitive intervention to prevent stroke.

A Emergency and initial management

Fulminant bleeding should first be controlled by immediate packing, with external auditory canal (EAC) packing and nasopharyngeal packing, if necessary, applied to achieve temporary haemostasis [4, 35]. This should be followed by urgent imaging, with immediate CT angiography to confirm the vessel course, localise the injury, and assess for pseudoaneurysm formation.

Packing alone can be considered only in cases with early and stable haemostasis, provided that intensive monitoring and immediate access to intervention are available, given the high risk of delayed pseudoaneurysm rupture or rebleeding. Jacobsson (1989) demonstrated that successful conservative management may require prolonged packing for up to 76 days, although re-bleeding remains a constant risk [15].

B Conservative vs. interventional/surgical management

Most reported cases required intervention (62.5% in the systematic review) due to pseudoaneurysm formation or rebleeding. Bathla et al. (2020) found a significant reduction in major complications with endovascular repair compared to ligation [4].

Surgical separation may be employed for symptomatic, uncomplicated a-ICA causing pulsatile tinnitus due to tympanic membrane or malleus contact, when hearing preservation is a priority. Successful resolution of tinnitus has been achieved by gently separating the tympanic membrane from the adherent vessel [8, 14].

Covered stent placement is an option for selected adult patients with poor collateral circulation, because it preserves ICA patency and may reduce stroke risk [35, 47]. However, this approach requires antiplatelet medication and carries a risk of in-stent thrombosis.

Surgical ligation or clipping is reserved for situations in which endovascular methods fail or are unavailable, or for irreparable injury [4, 35, 38, 41]. This approach carries a high risk of lasting neurological deficits and requires confirmation of adequate cerebral cross-circulation [13]. Higher complication rates have been reported with ligation than with endovascular approaches, with approximately 9% mortality [4].

C Management of intraoperative haemorrhage

Initial packing is used in 100% of iatrogenic injury cases and must be tight, though overpacking can damage the ICA [32, 35, 41]. In the Spörlein’s review, 6 out of 24 patients (25%) suffered neurological deficits including hemiparesis, aphasia, or minor facial weakness [35].

ICA occlusion or sacrifice is carried out via coiling, balloon, or ligation in 62.5% of cases requiring intervention [4, 33, 35]. Safety depends on adequate collateral cross-flow through the circle of Willis. Case #4 in the Spörlein’s series resulted in stroke due to traumatic occlusion [35].

## Conclusion

The reviewed literature indicates that preoperative recognition is the main safeguard in middle ear vascular anomalies. A true a-ICA, related to proximal ICA agenesis and collateral flow through Jacobson’s canal, must be separated from a lateralised petrous ICA, in which a normally calibrated artery is exposed at the genu by a dehiscent carotid plate. This distinction determines the operative risk. HRCT is the primary diagnostic test, and observation is usually safest unless symptoms, pseudoaneurysm, rupture or iatrogenic injury require treatment. When injury occurs, endovascular treatment is preferred when collateral circulation is adequate.

## Data Availability

The datasets used and analysed during the current study are available from the corresponding author upon reasonable request.
